# Supplementary material for: Targeted Metagenomic Databases Provide Improved Analysis of Microbiota Samples
Source: Microorganisms. 2024 Jan 10;12(1):135. doi: 10.3390/microorganisms12010135 (PMC10819777; doi:10.3390/microorganisms12010135)
Supplement: Supplementary file 1 [file microorganisms-12-00135-s001.zip › Figure S1.pdf]

Average species composition for the top 25 most abundant species, in **vagina flora - mother samples**

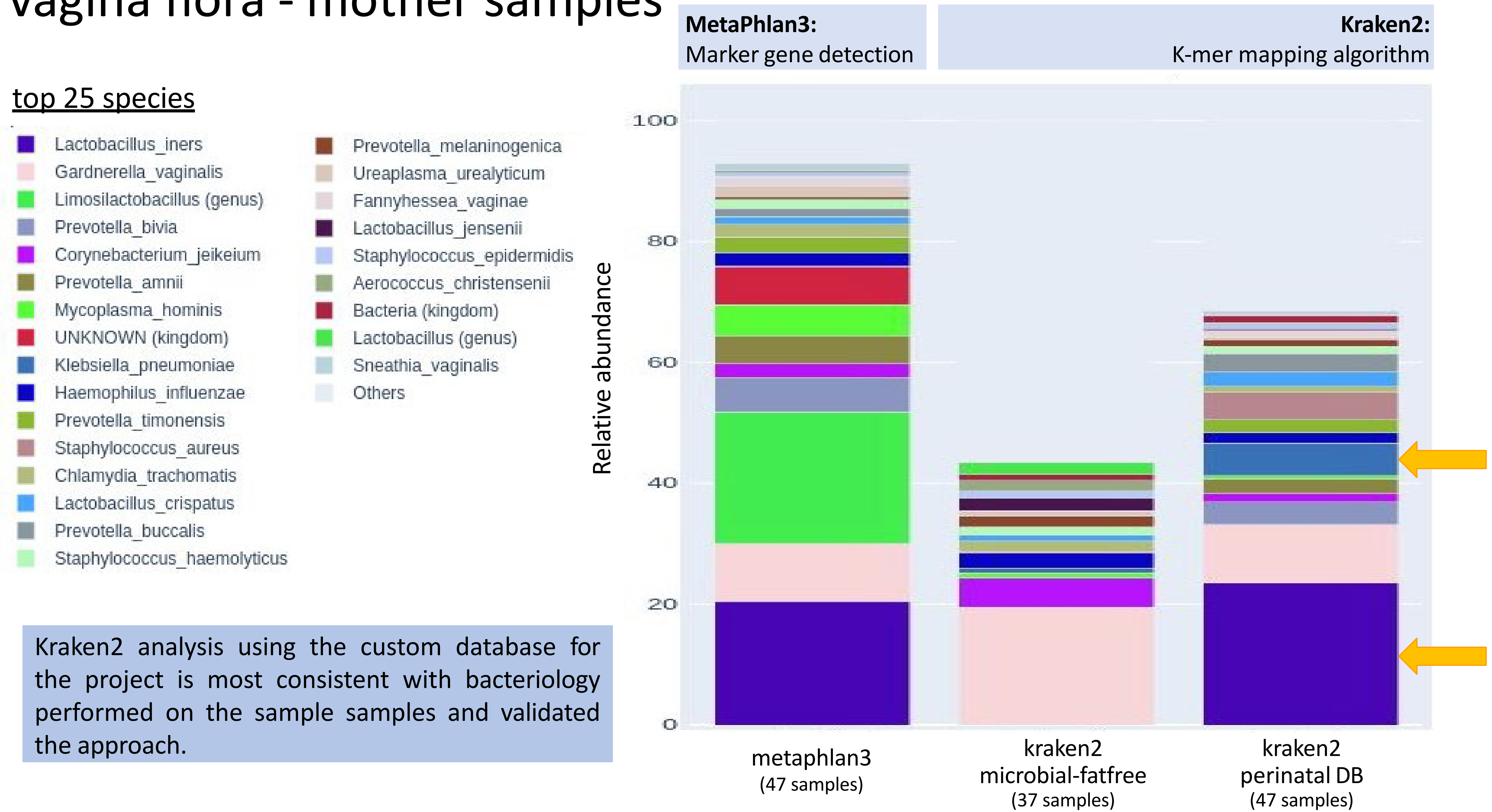

Kraken2 analysis using the custom database for the project is most consistent with bacteriology performed on the sample samples and validated the approach.
