# Supplementary material for: Targeted Metagenomic Databases Provide Improved Analysis of Microbiota Samples
Source: Microorganisms. 2024 Jan 10;12(1):135. doi: 10.3390/microorganisms12010135 (PMC10819777; doi:10.3390/microorganisms12010135)

Case: Blautia sp. CAG:257

Blautia genus

A

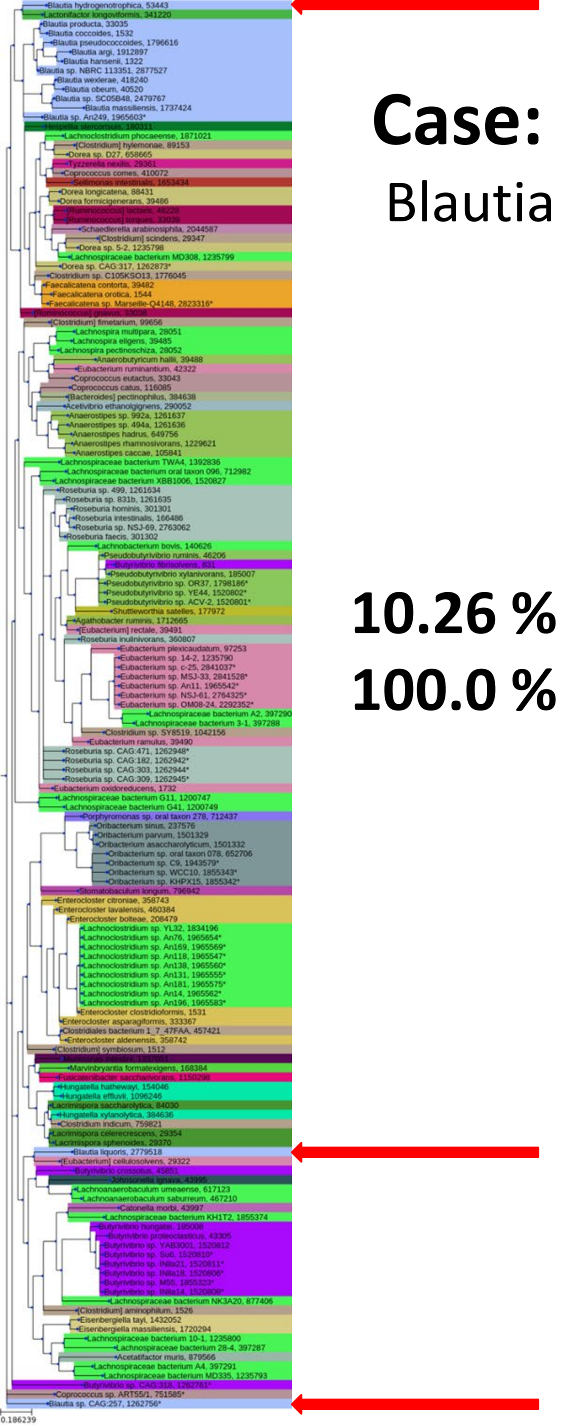

10.26 % Purity
100.0 % Completeness

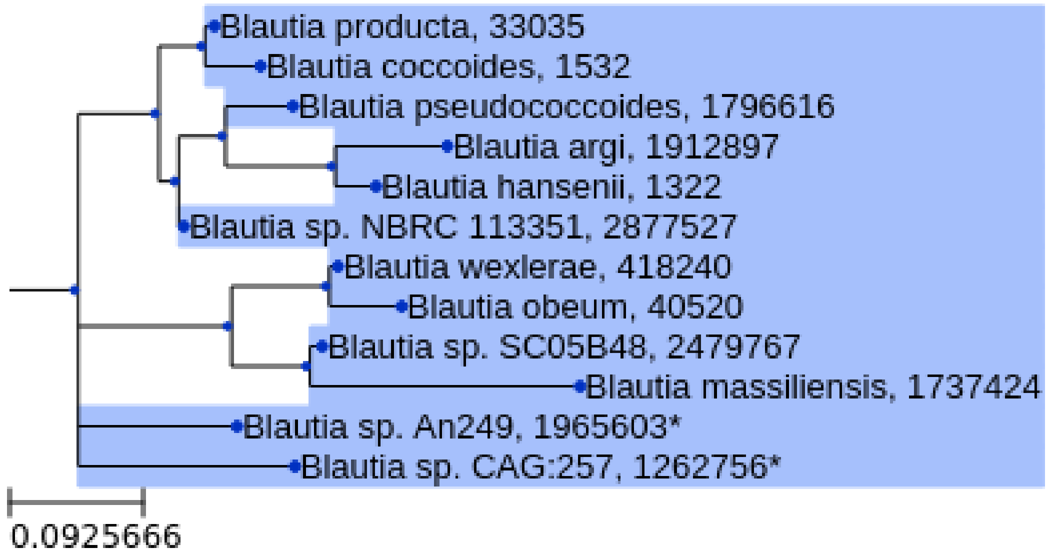

100.0 % Purity
83.33 % Completeness

Case: Peribacillus glennii & Peribacillus saganii

B

Peribacillus genus

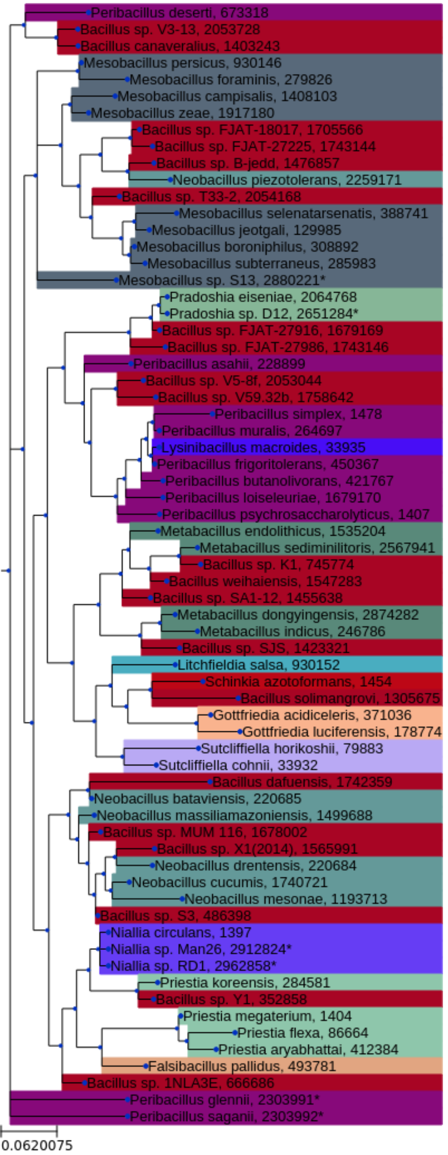

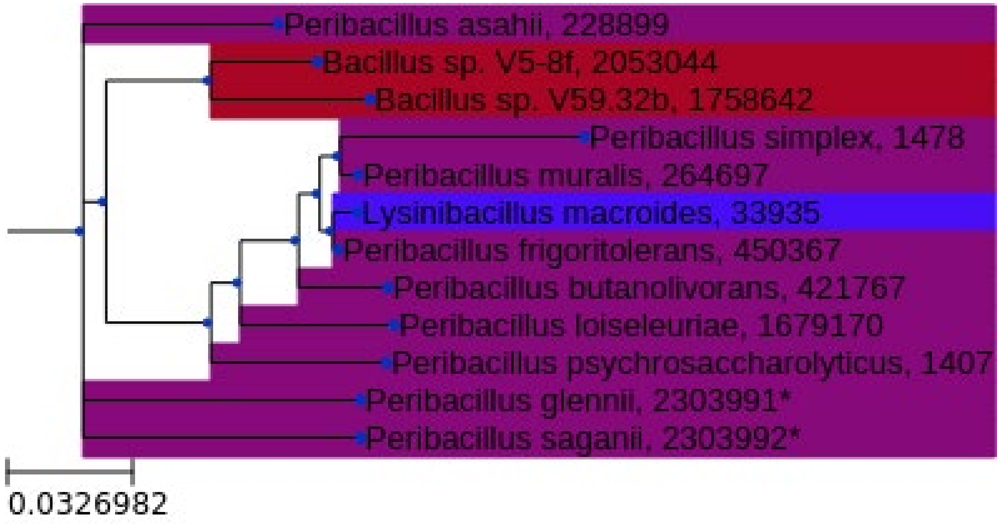

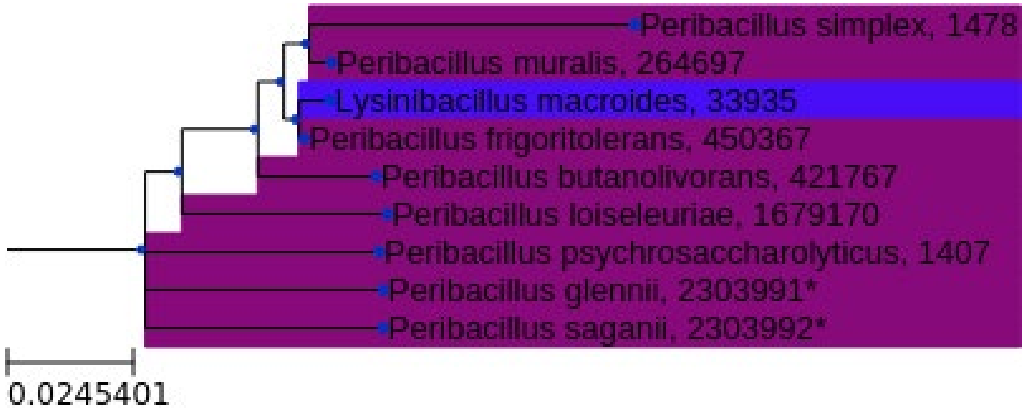

70.0 % Purity
87.5 % Completeness

85.71 % Purity
75.0 % Completeness

13.11 % Purity
100.0 % Completeness

Case : Citrobacter (species)

C

100 % of Citrobacter, 40% of the Citrobacter of the tree, no Citrobacter freundii

100 % of Citrobacter, 30% of the Citrobacter of the tree, Citrobacter freundii

75 % of Citrobacter, 60% of the Citrobacter of the tree, Citrobacter freundii

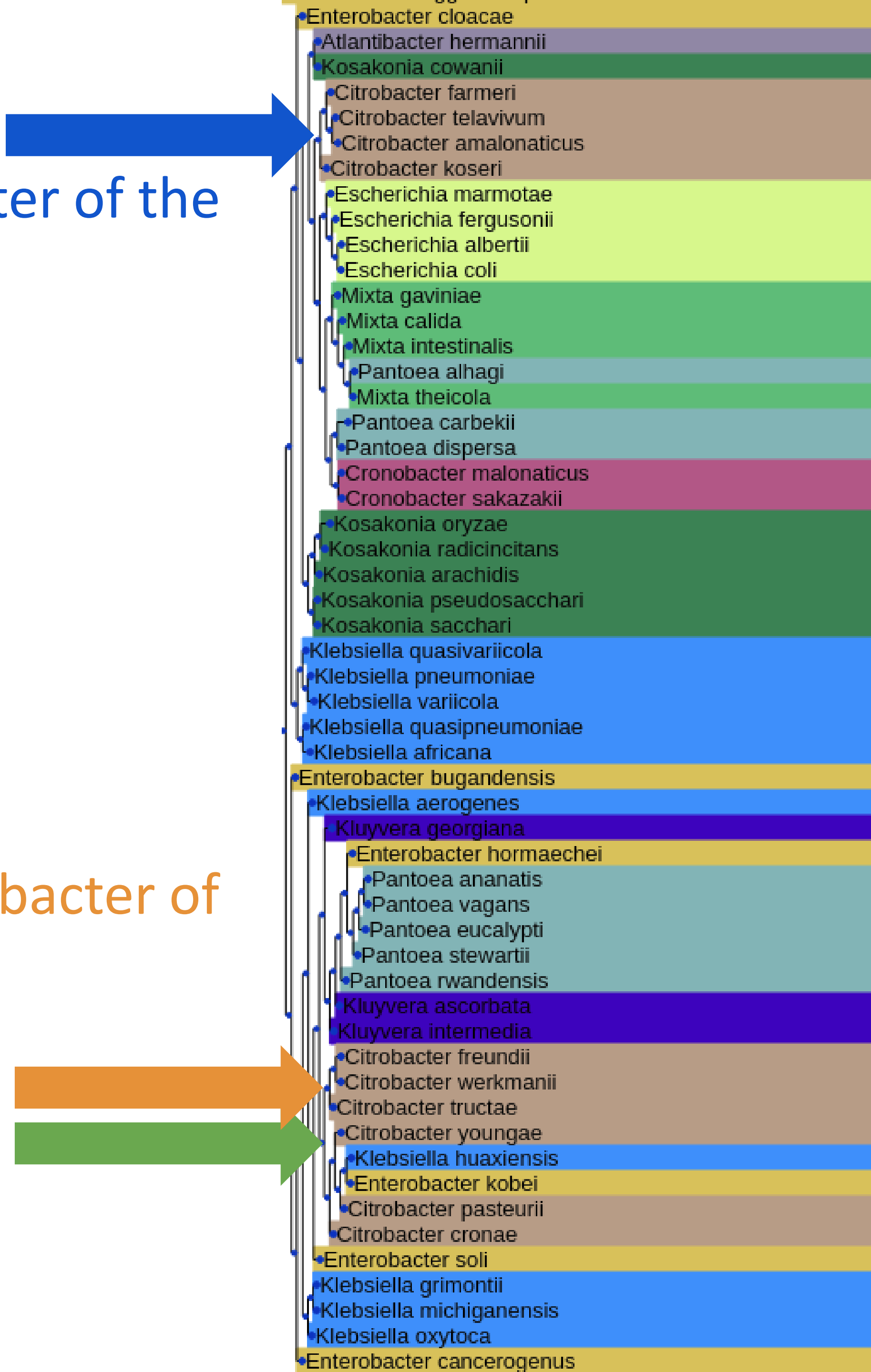

Supplement: Supplementary file 1 [file microorganisms-12-00135-s001.zip › Figure S2.pdf]
