# Supplementary material for: Targeted Metagenomic Databases Provide Improved Analysis of Microbiota Samples
Source: Microorganisms. 2024 Jan 10;12(1):135. doi: 10.3390/microorganisms12010135 (PMC10819777; doi:10.3390/microorganisms12010135)
Supplement: Supplementary file 1 [file microorganisms-12-00135-s001.zip › Supplemental Figure Legends.pdf]

Supplemental Figure 1: Comparison of species detection methods. An initial result comparing the identification of the 25 most prominent species found in the vaginal microbiota of a cohort of mothers. The list of species are on the left and the quantification in shown on the right. Quantification for methods in relative, with a maximum on 100%. Arrows show detection of *Lactobacillus* (purple), an important member of the healthy vaginal microbiota, and *Klebsiella* (blue-grey), a potential pathogen.

Supplemental Figure 2: Placement of missing taxa on phylogenetic tree. Illustration of the description in the Material & Methods section of how species without a published 16S rDNA rsequence were added to the phylogenetic distance tree used for diversity analysis. The cases illustrate how the a decision to maximize the sum of 'purity' and 'completeness' measurements were applied. (A) The illustration of the taxa *Blaitia* were a smaller subset of the tree was found to have significantly higher purity. (B) Example of *Peribacillus* genus where subsequent trimming of the tree used resulted in a small loss of completeness but a larger gain in purity. (C) Case of *Citrobacter* species where a maximization was useful in selecting the part of the tree among different regions with *Citrobacter* species.
